# Acknowledgement to Reviewers of *Journal of Cardiovascular Development and Disease* in 2018

**DOI:** 10.3390/jcdd6010004

**Published:** 2019-01-10

**Authors:** Journal of Cardiovascular Development and Disease Editorial Office

**Affiliations:** MDPI, St. Alban-Anlage 66, 4052 Basel, Switzerland

Rigorous peer-review is the corner-stone of high-quality academic publishing. The editorial team greatly appreciates the reviewers who contributed their knowledge and expertise to the journal’s editorial process over the past 12 months. In 2018, a total of 58 papers were published in the journal, with a median time to first decision of 14 days and a median time to publication of 36 days. The editors would like to express their sincere gratitude to the following reviewers for their cooperation and dedication in 2018:

Ahmad, ShakilLee, Dong IkAnderson, RobertLefkimmiatis, KonstantinosAránega, AmeliaLeung, AlanAronow, WilbertLevy, Finn OlavAsirvatham, Samuel J.Li, ShuaiAyme-Dietrich, EstelleLien, Ching-Ling (Ellen)Ballmann, ChristopherLin, Wey-jinqBeavo, JoeLorenzo González, ÓscarBelo, José AntónioLoughna, SiobhanBillaud, MarieLugnier, ClaireBlaser, MarkLui, Kathy O.Bolea, JuanMacquaide, NiallBorén, JanMaiellaro, IsabellaBraun, ThomasMänner, JörgBroderick, Tom L.Marín-Juez, RubénBroom, IainMaroteaux, LucBucchi, AndreaMcCoy, Sarah J. BreeseBurgon, Patrick G.McDaneld, Tara G.Butlin, MarkMcNamara, Coleen A.Campione, MarinaMeens, M.J.P.M.T.Campos-Toimil, ManuelMohamed, Salah A.Cao, JingliMolina, Cristina E.Capoccia, MassimoMorimoto, TatsuyaCedó, LídiaMoriya, ManabuCeulemans, BertenMovesesian, MatthewChaudhry, BillMyasoedova, VeronikaChen, Yung-ChihNakamura, KazufumiCiccone, Marco MatteoNaro, FabioCinnamon, YuvalNg, Fu SiongCurci, John A.Nikolaev, ViacheslavCzubryt, Michael P.Nishigaki, TakuyaDe Vecchis, RenatoO’Neill, LouisaDegenhardt, KarlPacurari, MaricicaDell’Acqua, MarkPaterson, David J.Dettman, Robert W.Postma, Alex V.Dimova, ElitsaPrakash, Siddharth K.Diviani, DarioPrickaerts, JosEichstaedt, ChristinaProtogerou, A.D.Esposito, FrancescoProvazník, IvoEzhov, Marat V.Puri, KritiFedele, FrancescoRocha, SoniaFetterman, Jessica L.Rodríguez-Pérez, José ManuelFranco, DiegoRubart, MichaelGenoni, AngelaRugonyi, SandraGerman, CharlesSacconi, LeonardoGittenberger-De Groot, Adriana C.Sampietro, TizianaGlover, MarkSander, VeronikaGrimes, Daniel T.Sang, Bing OngGubin, DenisSantulli, GaetanoGuo, Chao-YuSanyahumbi, Amy E.Gurha, PriyatanshSchmidt, MartinaHall, Kathryn T.Sedmera, DavidHarvey, RobertShewale, SwapnilHe, Jia-QiangShimada, YasuhitoHebert, TerryShiratori, HidetakaHiroi, NoboruSinzinger, HelmutHosick, PeterSivaguru, MayandiHouck, Philip D.Slark, JuliaHouston, Mark C.Smyth, JamesHuang, ChristopherSong, SuIkonomidou, Vasiliki N.Staples, AnneIntapad, SuttiraStrychalski, WandaIvanovitch, Kenzo D.Sun, YunfuIwasa, ToruTalman, VirpiJadhav, Gopal P.Tanaka, ToshihiroJanus, EdwardTritapepe, LuigiJaźwińska, AnnaVerhoeven, AdrieJeong, In CheolWard, Marie-LouiseJeremy, RichmondWatanabe, EiichiJouk, Pierre SimonWatanabe, MichikoKang, JunsuWatts, GeraldKardassis, DimitrisWeninger, Wolfgang J.Karra, RaviWesson, DonKassiri, ZamanehWinkler, JohannesKawashima, TomokazuWon, KimberlyKelly, Robert G.Wu, MingfuKing, BenjaminYamada, ShigehitoKlussmann, EnnoYamagishi, HiroyukiKocica, Mladen J.Yanagisawa, SatoshiKoga, Jun-ichiroYarwood, StephenKostner, GerhardYeh, Jwu-LaiKostner, KaramYin, VootKumar, SandeepYoshimura, MasamiLang, DiZoltowska, Dominika M.Lazzeri, Chiara


